# Attenuation of Stress Responses to Human Handling Through Habituation in Goats

**DOI:** 10.3390/ani15101385

**Published:** 2025-05-10

**Authors:** Tharun Tej Erukulla, Phaneendra Batchu, Priyanka Gurrapu, Arshad Shaik, Thomas H. Terrill, Govind Kannan

**Affiliations:** Agricultural Research Station, Fort Valley State University, Fort Valley, GA 31030, USA; terukull@widcat.fvsu.edu (T.T.E.); phaneendra.batchu@fvsu.edu (P.B.); priyanka.gurrapu@fvsu.edu (P.G.); arshad.shaik@fvsu.edu (A.S.); terrillt@fvsu.edu (T.H.T.)

**Keywords:** behavior, goats, habituation to handling, metabolomics, stress physiology

## Abstract

Goats raised on pastures are seldom handled except for purposes such as weighing and providing veterinary care. Regular positive interactions with human caretakers have been reported to attenuate fear and stress responses to routine human handling; however, this has not been adequately studied in meat goats. This experiment was conducted to determine the effects of habituation to handling on behavioral, physiological, and metabolomic responses in goats when subjected to routine handling. Seventy-two male (uncastrated) Spanish goats (6 mo; BW = 25.2 ± 0.37 kg) were randomly allocated to one of two treatment (Trt) groups: (i) regularly handled by stroking the back of each goat before feeding time for 90 days (handled: H) or (ii) not subjected to handling during the same period, but all other conditions were the same (non-handled: NH). After the 90-day habituation period, the goats were subjected to an arena test in the presence of an observer. Immediately after the test, the goats were subjected to routine veterinary exams that involved blood sampling (0 min) and measuring heart rate (HR), respiratory rate (RR), rectal temperature (RT), and body weight (BW). Blood samples were also collected after the veterinary exam (20 min) to determine physiological and metabolomic responses. Data from the arena test were analyzed using a Mann–Whitney U Test, and blood physiological responses were analyzed using MIXED procedures in SAS with sampling time (Time) as a repeated measure. Unpaired *t*-tests showed that the increase in BW was higher in the H goats (*p* < 0.01) compared to the NH goats over the 90 d habituation period. The approach distance from the observer (*p* < 0.05) and frequency of urination (*p* < 0.05) were greater in the NH goats during the arena test. HR (*p* < 0.01) and RR (*p* < 0.01) were higher in the NH group compared to the H group. Plasma cortisol concentrations were higher at 20 min than at 0 min (*p* < 0.05), while epinephrine (*p* < 0.05), metanephrine, normetanephrine, phenylethylamine, and 5-methoxytryptamine concentrations were higher (*p* < 0.05) in the NH goats than in the H goats. A targeted metabolomics analysis showed that six of the eight affected amino acids were lower (*p* < 0.05), and six of the seven affected phosphatidylcholines were higher (*p* < 0.05) in the NH goats compared to the H goats. The results show that habituation reduces fear and stress responses to routine handling, in addition to increasing BW, which can improve the welfare of meat goats.

## Simple Summary

Meat goats typically have minimal human interaction. Animals not accustomed to human handling can experience fear and stress when handling and restraint become inevitable. The beneficial effects of gentle human–animal interactions on animal welfare have been well documented; however, studies on meat goats are limited. In this study, seventy-two male Spanish goats were allotted to one of two treatment groups. Goats in one group were regularly handled (H) by stroking before feeding time every day for 90 days, while those in the other group were not subjected to handling (NH), but all other conditions were the same. An arena test conducted in sets of three goats with an observer seated in the pen showed that the H goats approached the observer more closely than the NH goats. The NH goats urinated more frequently than the H goats during the arena test, indicating fearfulness. Heart rate and respiratory rates measured after the arena test were also higher in the NH goats compared to the H goats. After the 90-day handling period, the increase in body weight was higher in the H goats than in the NH goats. Concentrations of several catecholamines in the blood were elevated in the NH goats, indicating higher stress levels than in the H goats. Metabolomics profiling also revealed perturbances of several metabolites indicative of elevated stress in NH goats. The results indicate that regular gentle handling of goats can reduce fear and stress responses during routine management procedures.

## 1. Introduction

Farm animals are exposed to human interactions for varying degrees and purposes, depending on the production system involved, which can cause fear and stress responses. Animals in intensive production systems are likely more accustomed to human handling than those raised outdoors on pasture or rangeland. In highly automated production establishments, human–animal interactions are rare, except during stressful or painful management procedures [1]. These include events such as weighing, blood and fecal sampling, veterinary examinations, and other procedures.

Meat goats are no exception in experiencing fear and stress due to human approach and handling, particularly when not accustomed. Such procedures invariably evoke stress responses in goats due to the disturbance to their homeostasis if the animal perceives the interaction as a negative experience and regards humans as a threat [2]. However, this perception can be changed when goats are exposed to regular, positive interactions with humans, which can improve animal welfare [3]. Habituating animals to handling, in addition to improving animal welfare, can consequently improve their health and productivity [4,5]. Therefore, care should be exercised during human–animal interactions for habituation to humans to occur and prevent animals from displaying fearful responses during routine, inevitable management-related encounters with humans. Developing human competency in animal handling is imperative in minimizing handling-related stress in animals [6].

Subjecting goats to the same handling treatment repeatedly can reduce their flight distances in due course. Researchers have studied several methods of habituating animals to human approach and handling, from caretakers regularly walking amongst the herd [7] to maintaining frequent tactile contact [8]. Lensink et al. [9] showed that positive interactions during feeding were associated with ease of handling, growth, and meat quality in veal calves. In goats, regularly providing feed inside a livestock trailer habituates them to the trailer environment and reduces stress responses during long-duration transportation [10,11].

Several methods have been studied to evaluate fear and stress responses to humans and handling, including behavioral observations and physiological measures. Behavioral tests, such as approach tests, combined with blood cortisol and catecholamine concentrations, can indicate fear, stress, and distress levels in goats. For instance, Pascual-Alonso et al. [12] reported that lambs experiencing gentle human contact had lower cortisol levels than the controls. Because the metabolome is highly sensitive to disturbances in homeostasis, a metabolomics approach can provide the most direct evidence of an animal’s physiological status [13]. However, the use of metabolomics, a large-scale study of low molecular weight molecules, as a tool to evaluate animal welfare has been explored little, although it is a promising approach [11]. Studies in goats have indicated that stress can significantly alter their blood metabolome [11,14]. Several blood amino acid levels were lower, and long-chain acylcarnitine concentrations were higher due to transportation in goats that were not previously habituated to livestock trailers compared to those fed regularly inside the trailer [10].

Goats raised for meat production are typically not confined, but when inevitably handled for routine management or veterinary care, they invariably experience fear and stress that can compromise their welfare, in addition to making the handling task harder. However, the effects of human handling on stress in meat goats are still poorly understood. Therefore, the objective of this experiment was to determine the effects of regular handling of Spanish goats at feeding time on their behavioral, physiological, and metabolomic responses when subsequently exposed to routine management-related handling and veterinary exams.

## 2. Materials and Methods

### 2.1. Animals

The animal care protocol for this study was reviewed following the ADSA-ASAS-PSA Guide for Care and Use of Agricultural Animals in Research and Teaching [15] and approved by the Institutional Animal Care and Use Committee at Fort Valley State University. Goat kids purchased from a farmer were used for this experiment. After the quarantine period, the goats were dewormed two weeks prior to the beginning of the trial. Each goat was already ear-tagged for identification at the farm of origin.

### 2.2. Experimental Procedure

**Figure 1:**
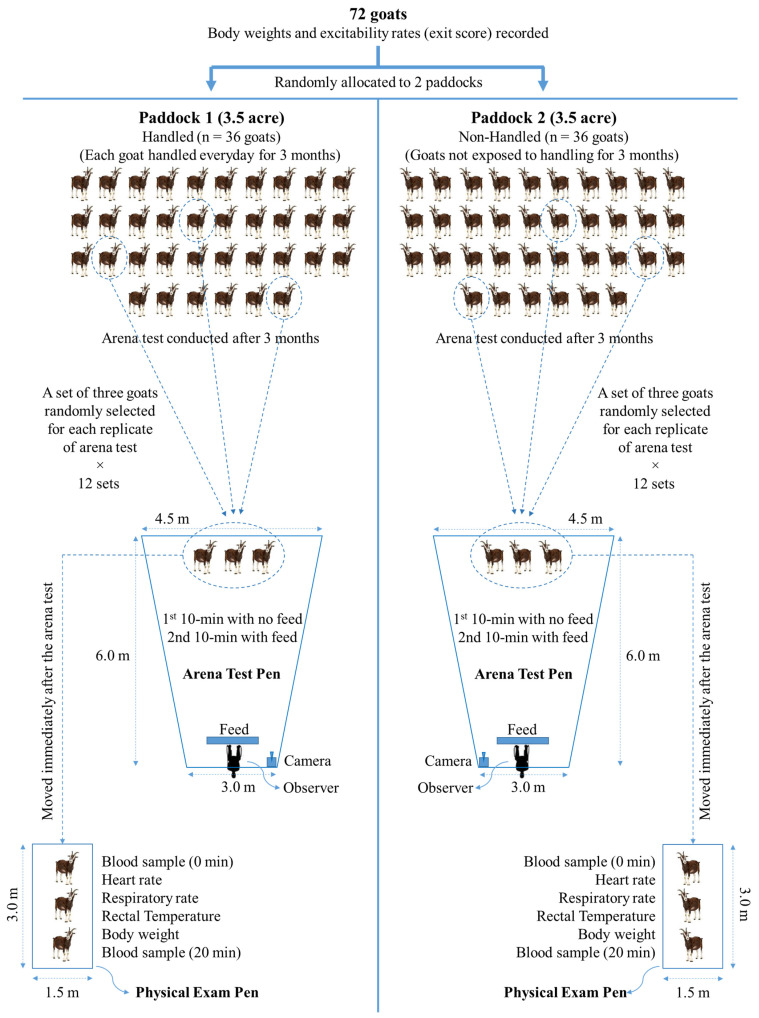
The allotment of goats to the Trt groups and the shape and dimension of pens for arena testing and physical examination. This figure does not represent the actual location of the arena test pens.

For this study, a total of 72 six-month-old uncastrated male Spanish goats (BW = 25.2 ± 0.37 kg) were individually weighed, rated for excitability by recording an exit score, and allocated to one of two identical 3.5-acre paddocks, with each paddock representing one of the two treatments (Trts, Figure 1). Goats in Paddock 1 were regularly handled every day during feeding time for 90 days (handled: H), and those in Paddock 2 were not subjected to handling during the 90-day period (non-handled: NH), but all other conditions were identical. The 90-day habituation period was chosen to ensure the animals had sufficient human interaction, although previous studies have shown that meat goats can become habituated to a procedure in a much shorter duration [11]. Animals were allowed to graze on natural pasture primarily comprising Bermudagrass (*Cynodon dactylon*) with ad libitum access to hay and water. Goats were also provided with a concentrate supplement (commercial goat pellet; 18% crude protein) in a feeding pen (9 × 6 m) within each paddock, containing linear feeders with sufficient feeder space (0.27 m/goat) for all goats to access feed without competition. The feeding pens were established at the opposite ends of the two rectangular paddocks so the NH goats could not see the H goats being subjected to regular handling.

### 2.3. Excitability Rate

To determine whether the excitability rate of an animal has any effect on its ability to be habituated to regular handling, exit scores (ESs) were recorded when the goats were weighed for the first time under the assumption that the temperaments of individual goats do not change over time. Previous reports have shown that temperament scores are stable over time in livestock animal species [16]. Studies on goats also support the point that the temperament is consistent over time. Lyons [17] reported that with respect to reaction to humans, goats express consistent individual differences in temperament. Finkemeier et al. [18] concluded from their experiment involving Nigerian dwarf goats that individual variations in goat personalities are consistent over time and opined that personality should be taken into account when introducing management changes to improve animal welfare. Temperament is a personality trait. Goats were scored based on their behavior when they exited the weighing chute according to the procedure described by Vetters et al. [19]. A score of 1 was given if the animal walked, 2 if it trotted, 3 if it cantered, and a score of 4 if the goat ran out of the chute. Three observers independently recorded their scores, and the average was taken as the ES of the animal. The inter-observer reliability was good (Weighted Kappa ± SEM = 0.73 ± 0.071).

### 2.4. Handling Treatment

The same three individuals (handlers) started the feeding routine every day at a set time (9:00 a.m.), alternating the order between Trts every day. During feeding time, all goats entered the feeding pen voluntarily. Every day, once the H goats entered the feeding pen, the gate was closed so that every goat could be subjected to handling. Further, the feeding pen for the H goats was divided into two smaller pens with movable panels to reduce the size of the area and facilitate the handling process without chasing the goats around to catch them for handling. Each handler approached the goat deliberately so that visual contact was made first, and then gently restrained the goat and softly stroked it on the back of its body for approximately 5 s. The handling pen was small enough that the goats stayed put with minimum movement as the handlers approached, restrained, and completed the handling routine every day with ease. Movement of goats prior to gentle restraint, when noticed, ceased immediately after the tender stroking started. All goats stood on four legs during the handling process. Each animal was handled for approximately 5 s once every day by one of the handlers, with all three handlers using a consistent, gentle handling procedure. After all animals were handled, they were allowed access to feed in the other half of the feeding pen. The pen gate was opened after handling so that the goats could go free in the paddock after feeding. The NH goats were subjected to the same feeding procedure, except the goats were not handled, and the handlers left the feeding area and the paddock immediately after providing feed.

### 2.5. Arena Test

After the 90-day handling period, goats were subjected to the arena test. The test pens were built using portable metal grilled panels adjacent to the feeding pens in the respective paddocks, which were locations the goats were familiar with. These locations were presumed to avoid the confounding effect of location unfamiliarity on the effect of the Trt. To avoid the effect of social isolation, three goats were tested at one time in sets rather than individually (3 goats/set; 12 sets/Trt), as shown in Figure 1. The arena tests were conducted on consecutive days over a period of six weeks, with 2 sets being tested from each Trt group every day. The order of application of arena tests was alternated daily between the treatment groups. The 20 min arena test comprised a pen with three randomly chosen goats from within one of the Trt groups and an observer seated behind a linear feeder at one end. For each test, the three goats were led into the pen with the observer already seated in his position. During the first 10 min period of the test, the feeder was kept empty, and during the second 10 min period, the feed was added to the feeder so that the three goats could see the feed. Behavior observations were recorded using a video camera as well as in real-time by the observer in the pen and by two other observers from a point approximately 20 m from the pen, so as not to distract the test subjects. A GoPro Hero8 camera (Woodman Labs, Inc., San Mateo, CA, USA) was mounted close to the observer in each pen to record the behaviors. For easy monitoring of the movement and location of goats within the test pen, lines were drawn on the ground using spray paint every meter to demarcate the distances.

### 2.6. Behavioral Observations

Behaviors recorded on individual goats included latency to approach (LA), approach distance (AD), and frequencies of agonistic encounters (AE), urination, defecation, and escape attempts. The three observers replayed the video recordings independently to verify and confirm observations each of them made in real time during the arena tests. The behavioral observations by the three observers were then averaged to arrive at the data points used for statistical analysis. Latency to approach was recorded for individual subjects as the time interval in seconds from when the goat was placed in the test pen to the first step it took toward the observer, both with and without feed in the feeder. Approach distance was recorded for each goat as the closest distance between the observer and the goat with and without feed. The frequency of agonistic encounters was recorded as the encounters each goat was involved in, regardless of whether or not it initiated the interaction, both with and without access to feed. Frequencies of urination and defecation were recorded every time the goat urinated or defecated. When a goat lifted its front legs, standing close to and facing the pen panel, it was recorded as an escape behavior.

### 2.7. Physical Exam and Blood Sampling

Immediately following the arena test, the pen gate was opened, and the three goats were led into a small pen (3.0 × 1.5 m), built using the same type of panels used for the arena test pen, for a post-treatment handling that simulated a routine veterinary physical exam. As soon as the goats entered the physical exam pen, blood samples were collected (0 min), and the heart rate, respiratory rate, rectal temperature, and body weight of individual subjects were measured. Exactly 20 min after the 0 min sampling, another blood sample was taken from each animal (20 min sample) in the same order, followed by the 0 min sampling. A 20 min period was allowed between the two blood samples to determine the effect of routine handling on stress hormones and metabolites, since, for example, it takes approximately 15 min for blood cortisol to increase after a stressor. Cortisol is accepted as a reliable indicator of stress in goats.

Blood samples were collected by jugular venipuncture into disposable vacutainer tubes containing 81 µL of 15% EDTA solution. The blood tubes were placed on ice until the separation of plasma. The samples were centrifuged at 1000× *g* for 20 min; plasma was pipetted into different aliquots and then stored at −80 °C until analysis.

Heart rate (HR) was measured by listening directly to the heartbeat using a stethoscope (for a more accurate measurement). The stethoscope was placed on the left side of the chest behind the front leg, and the number of beats over a period of 30 s was then multiplied by 2 to arrive at beats per minute (bpm). Respiratory rate (RR) was measured by counting the flanking breathing movements for 30 s, then multiplying it by two to obtain the breaths per minute. Rectal temperature (RT) was measured manually by inserting a digital thermometer approximately 2 cm into the rectum of each animal with the bulb in contact with the mucosa until the temperature stabilized.

### 2.8. Cortisol Analysis

A commercially available cortisol ELISA kit (Abnova, Taipei, Taiwan) was used to determine plasma cortisol concentrations following the instructions provided by the manufacturer and the steps outlined by Kannan et al. [11]. The minimum detectable concentration of cortisol using this method is 1.0 ng/m.

### 2.9. Catecholamine Analysis

All plasma samples were shipped on dry ice to the Metabolomics Innovation Center (TMIC) at the University of Alberta, Edmonton, Canada, for catecholamine analysis following the method described by Zheng et al. [20]. For profiling of catecholamines and other biogenic amines, a combined direct injection mass spectrometry with a reverse-phase LC–MS/MS custom assay using an ABSciex 4000 Qtrap tandem mass spectrometry instrument (Applied Biosystems/MDS Analytical Technologies, Foster City, CA, USA) with an Agilent 1260 series UHPLC system (Agilent Technologies, Palo Alto, CA, USA) was used. Analyst 1.6.2. was used for data analysis.

### 2.10. Targeted Metabolomics Analysis

The metabolomics analysis was also conducted at the TMIC. A targeted quantitative metabolomics technique that combined direct injection (DI) mass spectrometry with a reverse-phase LC–MS/MS custom assay was used to analyze all the samples. Up to 150 different endogenous metabolites, including amino acids, acylcarnitines, biogenic amines and derivatives, uremic toxins, glycerophospholipids, sphingolipids, and sugars, were identified and quantified using this custom assay combined with a mass spectrometer. In this approach, derivatization and extraction of analytes were combined with selective mass spectrometric detection using multiple reaction monitoring (MRM) pairs.

The samples were thawed on ice, vortexed, and centrifuged at 13,000× *g* for all metabolites except organic acids. The center of the filter on the upper 96-well plate was loaded with 10 µL of each sample, dried in a nitrogen stream, and then phenylisothiocyanate was added for derivatization. After incubation, the filter spots were dried again with an evaporator. The metabolites were extracted using 300 µL of extraction solvent. The extracts from centrifugation into the lower 96-deep well plate were then diluted with MS running solvent. For organic acid analysis, 50 µL of the sample was mixed with 150 µL of ice-cold methanol and 10 L of isotope-labeled internal standard mixture. After overnight protein precipitation, it was centrifuged for 20 min at 13,000× *g*. A 50 µL supernatant was loaded into a 96-deep well plate, followed by the addition of 3-nitrophenylhydrazine (NPH) reagent. After a 2 h incubation, the BHT stabilizer and water were added before LC-MS injection.

For mass spectrometric analysis, an ABSciex 4000 Qtrap^®^ tandem mass spectrometry instrument (Applied 03 Biosystems/MDS Analytical Technologies, Foster City, CA, USA) with an Agilent 1260 series UHPLC system (Agilent Technologies, Palo Alto, CA, USA) was used. An LC approach was used to deliver the samples to the mass spectrometer, followed by the DI method. The data were analyzed using Analyst 1.6.2.

### 2.11. Statistical Analyses

#### 2.11.1. Behavioral Responses

The behavioral data were not normally distributed; therefore, a Mann–Whitney U Test (non-parametric) was used in SAS to analyze the difference between the H and NH groups. Since three goats were used in each set for the arena test, the set was taken as the experimental unit, and the average values for the three goats were taken as data points. Since the goats rarely showed escape and defecation behaviors, these variables were dropped from the analysis. Pearson correlation analysis was conducted using SAS version 9.4 to study the association between ESs and the behavioral variables studied. However, the individual animal was treated as the experimental unit for correlation analysis among the behavioral variables and physiological stress indicators.

#### 2.11.2. Body Weight and Physiological Stress Responses

The increase in body weight, HR, RR, and RT data was analyzed using an unpaired *t*-test in SAS to determine differences between the H and NH treatment groups. The increase in BW over the 90-day period was calculated by subtracting the weight taken before the beginning of handling treatment from that taken after the arena test. Plasma cortisol, glucose, epinephrine (EPI), norepinephrine (NorEPI), metanephrine (MN), normetanephrine (NorMN), dopamine, tyramine, phenylethylamine (PE), and 5-methoxytryptamine (5-MT) concentrations were analyzed using the MIXED procedures in SAS, with time as a repeated factor and animal as a random variable. The data were tested for normality and homogeneity of variance using Shapiro–Wilk’s Test and Levene’s Test, respectively, and appropriate data transformations were made when required. Graphs were created using the output from the interaction lsmeans, stderr, and pdiff options in SAS.

#### 2.11.3. Metabolomic Responses

The metabolomics data were treated in the following manner before analysis. Metabolites with identical concentrations for all samples and those with 20% of missing concentration were removed from the datasets. For the remaining metabolites with missing values, concentrations were replaced either with ½ of LODs (levels of detection) or 1/5 of the smallest metabolite concentration (the default Metaboanalyst’s imputation method), whichever was larger. For multivariate analysis, data were log-transformed and scaled by range scaling with Metaboanalyst R.

Because the data in all groups were not normally distributed, a comparison of two independent samples was conducted using the Mann–Whitney U rank method. For paired samples at different time points, the Wilcoxon test was performed. The false discovery rate (FDR) was obtained using the Benjamini–Hochberg method, the effect size was calculated using the Cliff’s Delta method [21,22], and fold change was determined by calculating the ratio between group medians. For pair-wise class comparisons, volcano plots were generated to show metabolites with concentration changes with *p*-values < 0.05 and fold changes above 1.3 or below 0.77. Two-way ANOVA and post hoc tests were conducted on log-transformed data using the Benjamini–Hochberg false discovery rate method to correct *p*-values for multiple comparisons.

Metaboanalyst R was used to perform principal component analysis (PCA) and partial least squares discriminant analysis (PLS-DA). The PLS-DA models were tested for performance and the absence of overtraining with 10-fold cross-validation. The model accuracy was considered satisfactory when R2 and Q2 were above 0.66. The model was considered not over-trained when R2 and Q2 were comparable with each other (i.e., within 20%). A permutation test was conducted to assess the statistical significance of the PLS-DA model, and a model was considered statistically significant if the *p*-value of the permutation test was below 0.05. Heatmaps were generated with Metaboanalyst 5.0 using default settings. An autoscaling was applied to concentration values prior to heatmap generation. For supervised clustering, the concentrations were grouped according to sample class (Trt or Time) in the sample dimension and using Euclidean distances and Ward’s method in the metabolite dimension of the heatmap.

## 3. Results

### 3.1. Behavior

**Figure 2:**
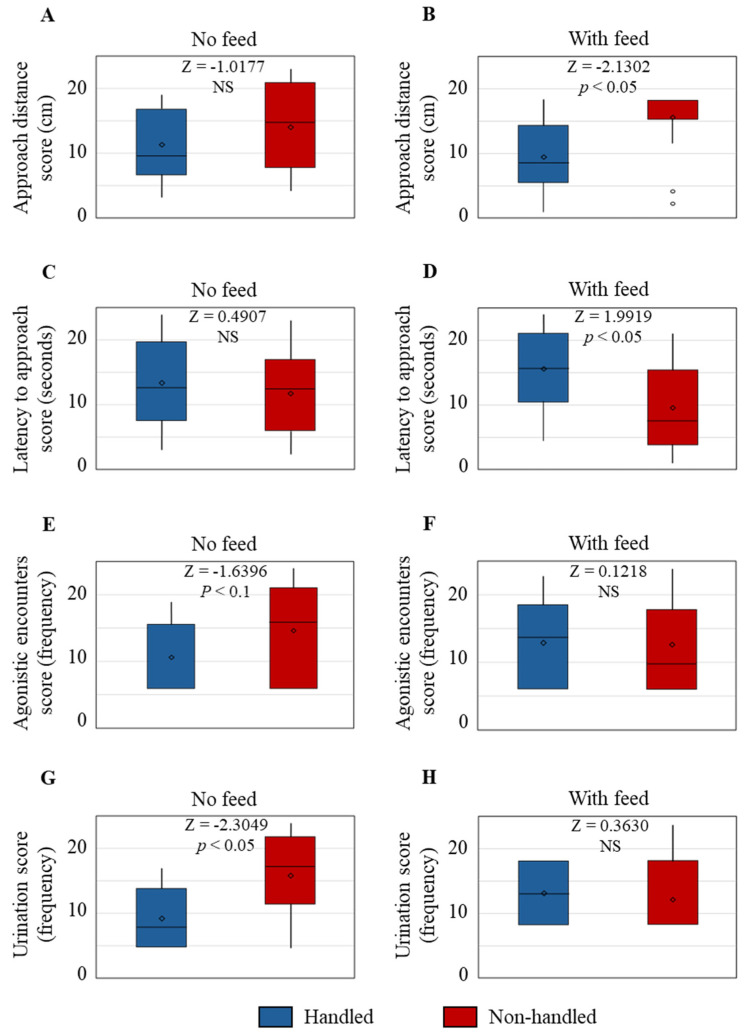
The distribution of rank scores (units of measurement shown in parenthesis on the Y axis) for (**A**) approach distance with no feed, (**B**) approach distance with feed, (**C**) latency to approach with no feed, (**D**) latency to approach with feed, frequencies of (**E**) agonistic encounters with no feed, (**F**) agonistic encounters with feed, (**G**) urination with no feed, and (**H**) urination with feed. The Z scores and probability levels shown are based on the Mann–Whitney U Test. NS = Not significant.

**Figure 3:**
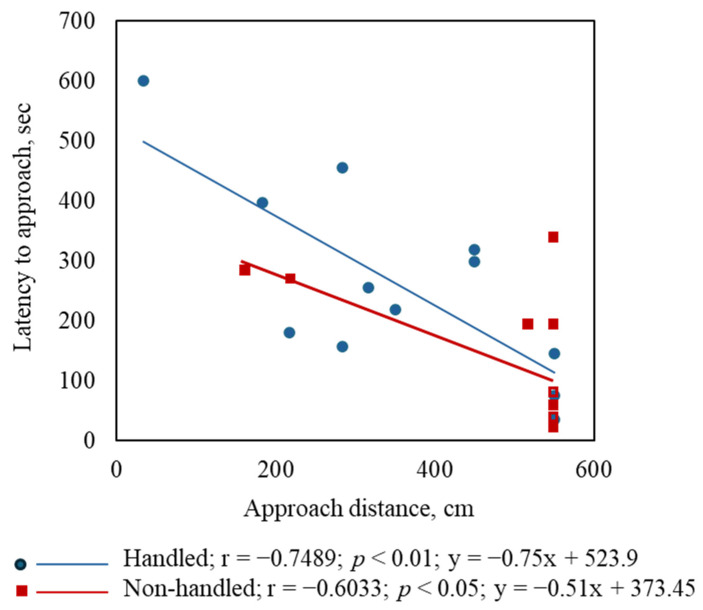
A scatter plot showing a significant negative correlation between latency to approach and approach distance in the two treatment groups.

Behaviors of the H and NH goats (averaged for three goats in each set) with and without feeding during the arena test are shown in Figure 2. While the AD was not different between Trts when there was no feed (Figure 2A), AD was significantly greater (*p* < 0.05) in the NH goats compared to the H goats when feed was provided (Figure 2B). However, while LA was not different between Trts during the first 10 min period (no feed; Figure 2C), LA was shorter in the NH group than the H group during the second 10 min period (with feed; Figure 2D). The frequency of AE was not significantly different between Trt groups with or without feed (Figure 2E,F), although there was a trend (*p* < 0.1) toward higher AE frequency in the NH group than in the H group during the first 10 min period (no feed). The frequency of urination was higher (*p* < 0.05) in the NH goats than in the H goats during the first 10 min period (Figure 2G). There was a significant negative correlation between LA and AD in both the H (*p* < 0.01) and NH (*p* < 0.05) groups (Figure 3).

### 3.2. Body Weight and Physiological Stress Indicators

**Figure 4:**
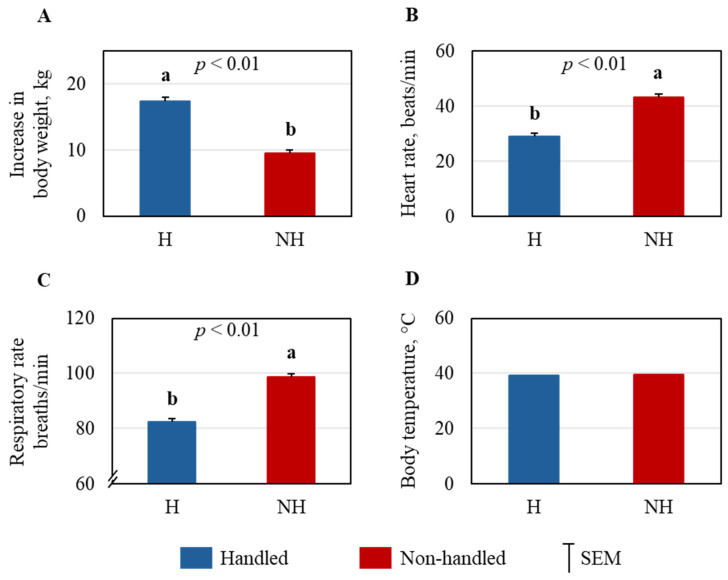
Effect of Trts (handled: H; non-handled: NH) on (**A**) increase in body weight after the three-month experimental period, (**B**) heart rate, (**C**) respiratory rate, and (**D**) body temperature. ^ab^ Means are significantly different at *p* < 0.01 as determined by *t*-test.

An unpaired *t*-test showed that the increase in body weight during the three-month handling period was significantly higher in the H goats compared to the NH goats (*p* < 0.01; Figure 4A). Both HR and RR were higher in the NH goats than in the H goats (*p* < 0.01; Figure 4B,C), while the RT was not affected by the handling treatment (Figure 4D).

**Figure 5:**
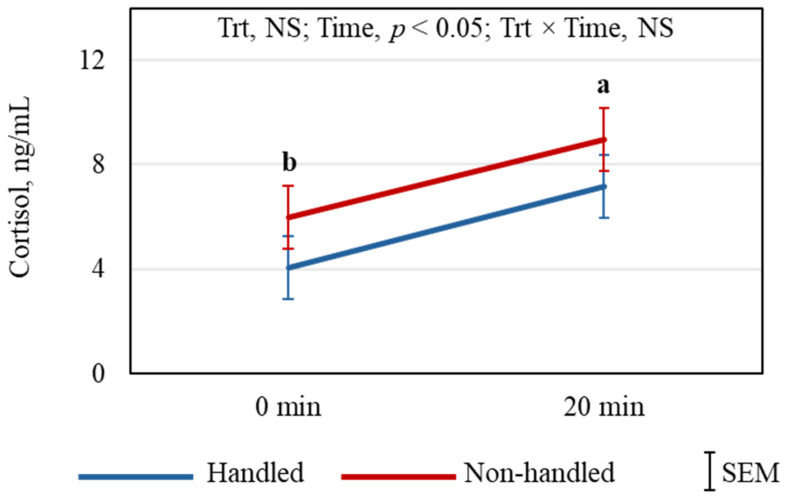
Effects of Trt, Time, and their interaction effects on plasma cortisol concentrations in goats. ^ab^ Time means are significantly different as determined by *t*-test at the indicated time point.

**Figure 6:**
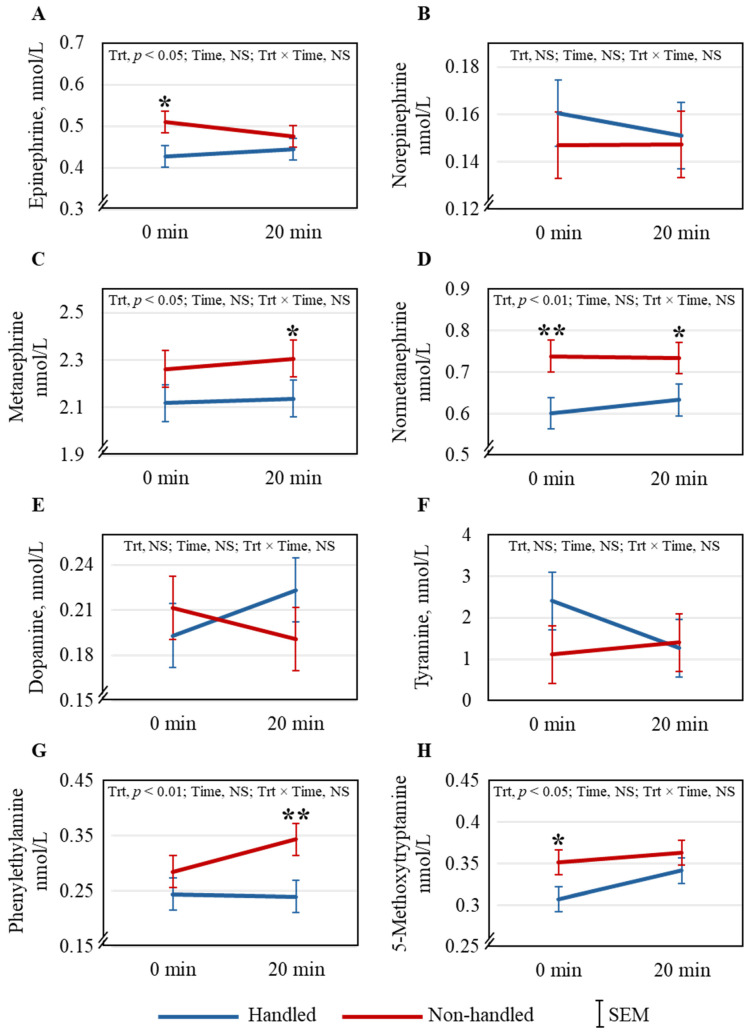
The effects of Trt and Time and their interaction effects on plasma (**A**) epinephrine, (**B**) norepinephrine, (**C**) metanephrine, (**D**) normetanephrine, (**E**) dopamine, (**F**) tyramine, (**G**) phenylethylamine, and (**H**) 5-methoxytryptamine concentrations in goats. * The treatment means are significantly different at *p* < 0.05, and ** indicates that the treatment means are different at *p* < 0.01 as determined by the pdiff procedure.

Routine veterinary exams of goats resulted in significantly increased plasma cortisol (*p* < 0.05) concentrations after 20 min compared to 0 min, while Trt did not have any effect (Figure 5). The effects of Trt, Time, and Trt × Time on the catecholamines studied are shown in Figure 6A–H. Treatment had significant effects on plasma EPI (*p* < 0.05), MN (*p* < 0.05), NorMN (*p* < 0.01), PE (*p* < 0.01), and 5-MT (*p* < 0.05) concentrations; however, Time and Trt × Time effects were not significant for any of the catecholamine concentrations. The results of the Pearson correlation analysis of behavioral and selected physiological responses of individual goats are shown in Table 1. There was a significant correlation between the excitability rates of goats and their plasma epinephrine concentrations immediately after the arena test (*p* < 0.001) in the NH group but not in the H group. The analysis confirmed the negative correlation between LA and AD recorded for individual goats in both H (*p* < 0.0001) and NH (*p* < 0.01) groups. There were negative correlations between AD and RR (*p* < 0.05) and between AD and RT (*p* < 0.05). There were also positive correlations between LA and HR (*p* < 0.05) and between LA and RT (*p* < 0.05).

### 3.3. Targeted Metabolomics

**Figure 7:**
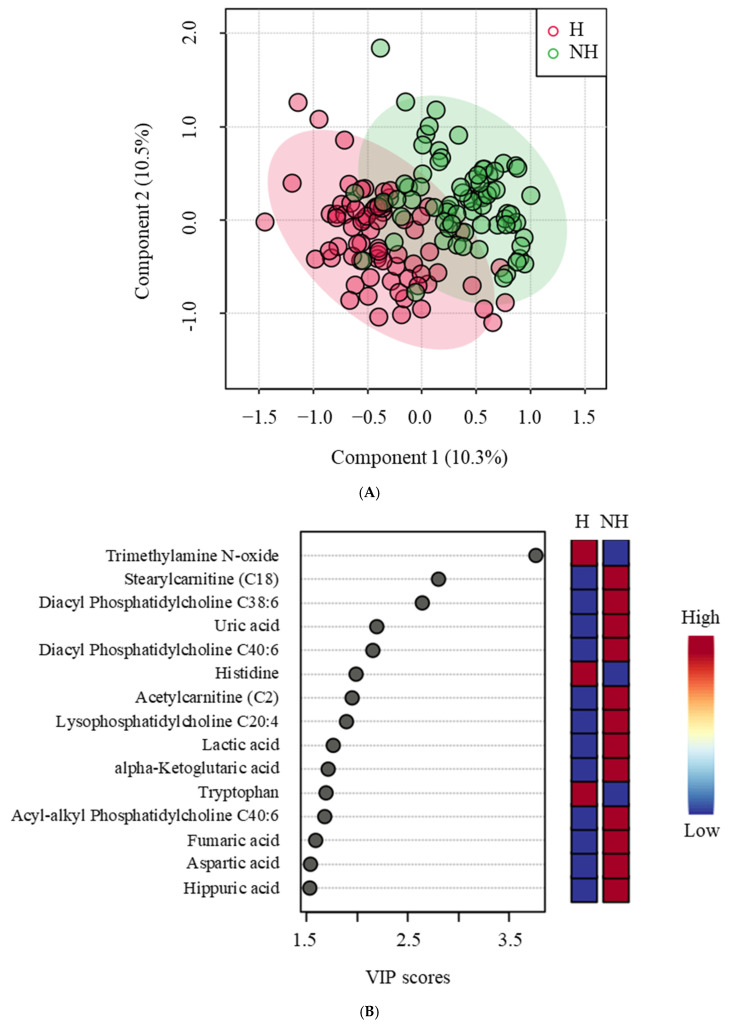
(**A**) The partial least squares discriminant analysis (PLS-DA) score plot of components 1 and 2 of Trts showing a modest separation of metabolites, and (**B**) a PLS-DA VIP plot showing the differences between the Trt (H = handled; NH = non-handled) groups and the metabolites (VIP scores > 1.5) that significantly contribute to the difference. The metabolite concentrations averaged across the two time points were used in the PLS-DA model (*p* < 0.05).

**Figure 8:**
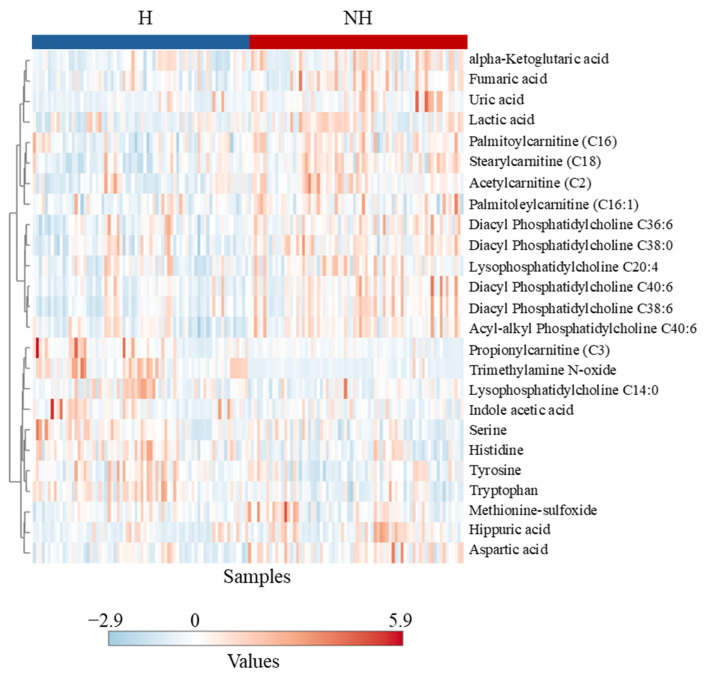
Hierarchical clustering heat map of plasma metabolites in goats in response to Trts.

Treatment effects with a *p*-value < 0.05 and non-negligible effect levels, along with fold changes, FDR, effect size, effect levels, and direction of change in group medians, are shown in Table 2. Trimethylamine N-oxide, PC aa C38:6, C18, PC aa C40:6, C2, uric acid, PC ae C40:6, PC aa C38:0, tryptophan, histidine, aspartic acid, PC aa C36:6, fumaric acid, C16, lysoPC a C20:4, lactic acid, C3, alpha-ketoglutaric acid, C16:1, arginine, and hippuric acid had statistically significant changes in concentrations based on both *p*-values and FDR (<0.05). Changes in the concentrations of tyrosine, serine, asparagine, citric acid, lysoPC a C14:0, glycine, and C0 had moderate statistical significance with *p*-values < 0.05 and FDR < 0.1. Additionally, aspartic acid, trimethylamine N-oxide, lactic acid, hippuric acid, uric acid, PC aa C38:6, C2, and C18 had *p*-values < 0.05 and fold changes above 1.3 or below 0.77. While PCA showed a modest separation between the H and NH groups, PLS-DA was able to separate the groups almost completely (Figure 7A). The PLS-DA model passed both cross-validation and permutation tests. Results of PLS-DA VIP analysis identified trimethylamine N-oxide, C18, PC aa C38:6, uric acid, PC aa C40:6, histidine, C2, lysoPC a C20:4, lactic acid, alpha-ketoglutaric acid, tryptophan, PC ae C40:6, fumaric acid, aspartic acid, and hippuric acid as significant for group separation (Figure 7B). These results were consistent with those of the univariate analysis. Unsupervised Ward’s clustering on the heatmap method partially separated the H and NH groups for the 25 metabolites with the lowest *p*-values (Figure 8).

**Figure 9:**
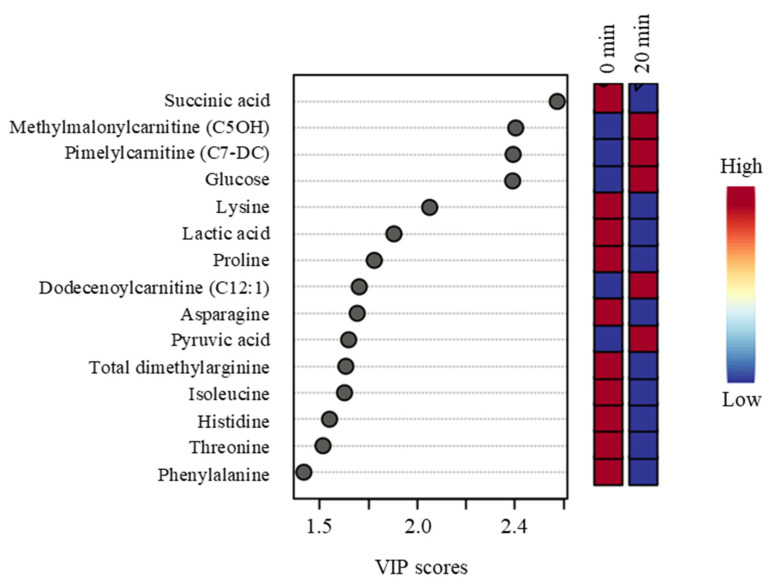
A PLS-DA VIP plot showing the differences among the sampling time groups (0 min and 20 min) and the metabolites (VIP scores > 1.5) that significantly contribute to the difference. The metabolite concentrations averaged across the two Trt groups were used in the PLS-DA model (*p* < 0.05).

Time effects with *p*-values < 0.05 and non-negligible effect levels, along with fold changes, FDR, effect size, effect levels, and direction of change in group medians, are shown in Table 3. Metabolites with significant changes (FDR ≤ 0.05) include succinic acid, lactic acid, lysine, asparagine, glucose, proline, threonine, pyruvic acid, serine, arginine, leucine, and isoleucine. Dimethylarginine and histidine had changes in the concentrations with moderate statistical significance (*p* < 0.05; FDR < 0.1). No metabolites had *p*-values < 0.05 and fold changes above 1.3 or below 0.77. Neither PCA nor PLS-DA could separate the different time points. The results of the PLS-DA VIP analysis show the metabolites that are significant for Time group separation (Figure 9).

**Figure 10:**
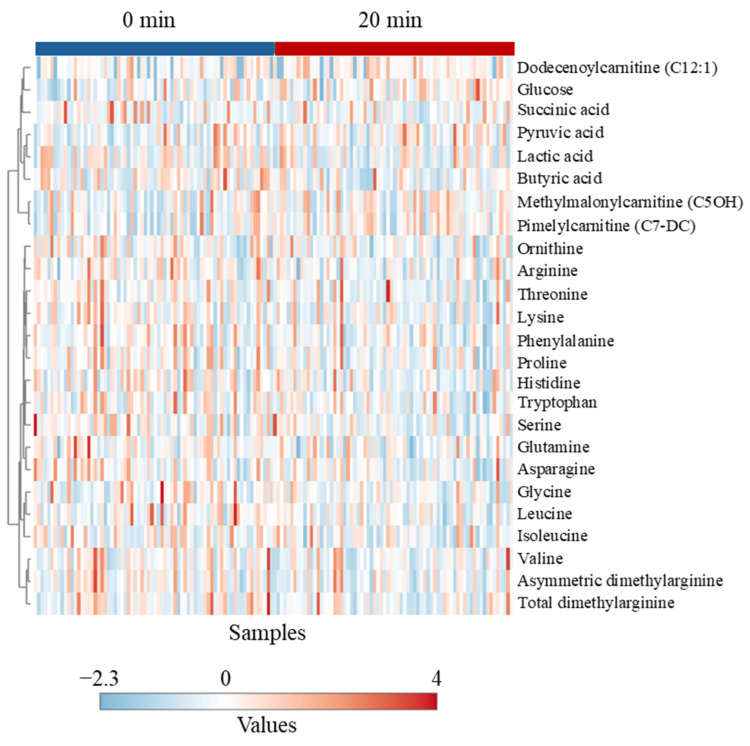
Hierarchical clustering heat map of plasma metabolites in goats in response to sampling time.

The unsupervised Ward’s clustering in the heatmap method could not separate the different time points (Figure 10).

A longitudinal analysis using two-way ANOVA showed that Trt × Time did not affect any of the metabolites. Therefore, interaction effects are not presented.

## 4. Discussion

Acclimatization of goats to human presence and handling is expected to reduce fear and excitement during occasions when handling is inevitable. Waiblinger et al. [23] identified three main categories of tests for assessing the animals’ responses to human beings, including reactions of animals to a stationary human, to a moving human, and to human handling. Two of these approaches—stationary human and human handling—were used in the present study to test the effects of habituation to handling on fear, behavior, and physiological responses.

Behavioral data collected during an arena test revealed that the AD was not different between the two Trt groups when feed was not presented. However, when feed was presented as a motivator, the AD from the observer was significantly greater in the NH goats compared to the H goats. The reluctance of NH goats to approach the observer voluntarily does not necessarily mean that the animals experienced a negative state or interaction [3]; however, in this experimental condition, the NH goats maintained a greater distance from the feeder due to the presence of the observer. While it is possible that the H goats approached the feed since the habituation treatment was imposed daily around feeding time and thus may have associated the observer with access to feed, it is also clear that the presence of the observer in the pen did not deter them from moving closer to the feed/observer than the NH goats, which is due to habituation to handling.

The LA was also not different between the Trt groups during the first 10 min period when feed was not presented, but the LA was shorter in the NH group than the H group during the second 10 min period after feed was added to the feeder. Even though the NH goats started moving toward the feeder/observer as soon as the feed was presented, they stopped their approach after the initial impulse and maintained a longer AD. The opposite effect seen in the H goats suggests that they did not respond to the addition of feed sooner than the NH goats, although they eventually started moving toward the feeder/observer, maintaining a shorter AD due to their habituation to human presence and regular handling. The negative correlation between AD and LA in both Trt groups when feed was presented further confirmed that the time taken for them to move toward the feed/observer did not necessarily indicate their response to human presence, but rather, it was their first reaction to the feed as a motivator. The results also indicate that AD more directly relates to response to human presence than LA under the conditions of this study.

Several studies have reported that gentle human interactions reduce aggressive episodes and agonistic encounters in farm animals, including poultry [24,25]. In our study, the frequency of AE was not significantly different between the Trt groups during the 20 min test period. However, there was a trend toward higher AE frequency in the NH group than the H group during the first 10 min period. Fear and psychological stress increase the frequency of micturition in mammals, which is considered an evolutionary predator defense mechanism [26,27]. In the present study, the NH goats urinated more frequently than the H goats, which indicates that the NH goats were more stressed and likely fearful.

Negative handling experiences on a regular basis have been reported to affect body weight gain in livestock animals. For example, Hemsworth et al. [28] found that young female pigs subjected to pleasant human contact for a 2 min period three times a week from 11 to 22 weeks of age had higher weight gain than those subjected to unpleasant human contact. A similar effect was also reported by Gonyou et al. [29] in pigs. In our study, the increase in body weight during the three-month handling period was higher in the H goats compared to the NH goats. Miller et al. [7] observed that average body mass increased over a three-week period in goats that had high human interaction compared to goats with low human interaction. In their study, the body mass increased in the low interaction group only during the first week of treatment but decreased thereafter, while the body mass steadily increased during the three-week period in the high interaction group and was significantly higher after the treatment period. It should be noted that in their study, goats were not handled in the high human interaction treatment, but rather the stockperson entered the pen and walked calmly among the goats for 20 min twice daily during the three-week experimental period, while the low human interaction group had no interaction other than during routine feeding and cleaning waterers. This indicates that regular positive interaction, even when there are no tactile contacts involved, is enough to see body weight gain in goats. In our study, weekly body weights were not taken to avoid any handling of the NH goats during the treatment period. The positive effect on weight gain in the H goats in our study may be due to less fighting among goats during feeding because of the presence of the three human handlers. Although not quantified, observations from a distance showed that the NH goats were more aggressive during feeding time, which could have prevented the weaker goats from accessing the feed, even though there was adequate linear feeder space allowance for each goat. Even during the arena test, there was a tendency for higher AE in the NH goats. Gentle human interactions have been reported to reduce agonistic encounters in farm animals [25].

In general, handling increases the heart rate and respiratory rate in goats not accustomed to human handling, and the extent of the increase can depend on the handler’s quality of handling and the training level. Preslaughter handling of goats by untrained handlers significantly increased the heart rate in goats, but not when handled by trained handlers [30]. Rough handling also increases the respiratory rate in goats, although the effects on rectal temperature are not significant [31]. In our study, both HR and RR were significantly higher in the NH goats than in the H goats, but the RT was not different between treatments. While the average HR in the H goats (82.4 ± 1.12 beats/min) was close to normal (normal range, 70–80 beats/min), the rate in the NH goats (98.6 ± 1.12 beats/min) was higher than normal. Both H and NH goats had higher than normal RR (normal range, 12–24 breaths/min); however, the RR in the NH goats was 43.3 (±0.99) breaths/min compared to the near-normal rate of 29.1 (±0.99) breaths/min in the H goats. The effects on HR and RR indicate that regular handling reduced excitement in goats during handling for routine blood sampling and veterinary physical exams. Coulon et al. [32] reported that lambs that were stroked had slower HR and higher root mean square of successive differences (RMSSD), which is considered to reflect parasympathetic reactivity and positive emotional state, compared to those that were not stroked [32]. However, Wang et al. [33] did not observe any difference in the HR due to the regular gentle handling of pigs, suggesting that HR response to habituation could be condition-specific and/or species-specific.

Habituation to handling did not differentially affect plasma cortisol concentration immediately following the arena test, but significantly increased in both Trt groups after the routine veterinary exam. Corticosteroid response to regular gentle handling is not consistent across studies in small ruminants [9] and poultry [34]. However, concentrations of several catecholamines and derivatives, including EPI, MN, NorMN, PE, and 5-MT, were significantly higher in the NH goats than in the H goats, indicating the NH goats were more stressed. Kadim et al. [35] reported increases in both EPI and NorEPI in small ruminants in response to stress; however, NorEPI was not different between the groups in our study. The increase in MN and NorMN, O-methylated metabolites of EPI and NorEPI, respectively, occurs when stress levels are elevated, and this has been reported previously in goats [14,27]. These breakdown products are suggested to be good indicators of stress in goats [14]. Phenylethylamine, produced in the mammalian brain in trace amounts, primarily in the dopaminergic areas [36], has been reported to modulate physical energy, attention, and mood [37]. During oxidative stress in rats, 5-MT could boost their antioxidant status because of its free-radical scavenging ability [38]. Adaptive behavioral responses to stress could be related to 5-MT concentrations in mammals [39]. If the responses to stress on these catecholamines are similar in goats, the lower PE and 5-MT concentrations in the NH goats could, therefore, indicate their lower emotional state than the H goats.

The excitability rates of individual goats assessed at the beginning of the trial were significantly correlated with their respective plasma epinephrine concentrations immediately after the arena test in the NH group but not in the H group. Curley et al. [40] reported that excitable calves had higher circulating catecholamine levels compared to calmer calves. Our results suggest that habituation can dilute the epinephrine reactivity and individual differences in excitability rates in goats. Individual animal AD values were inversely correlated with LA in both H and NH goats, confirming the results of correlation analysis with sets of three goats as experimental units.

Metabolomic profiling showed that of the eight amino acids significantly influenced by treatment, six were lower in the NH goats compared to the H goats. Histidine, arginine, serine, and asparagine are glucogenic in nature, and tryptophan and tyrosine are both glucogenic and ketogenic, all of which could have been used for gluconeogenesis in the NH goats. Decreased amino acid concentrations in blood could indicate an elevated rate of gluconeogenesis [41]. A similar effect was observed by Batchu et al. [10] in stressed goats, although in that study, the animals were challenged with intense and prolonged stress. The elevated levels of six of the seven significantly affected phosphatidylcholines and lyso-phosphatidylcholines noticed in the NH goats were likely associated with higher fatty acid metabolism in this group than in the H group. Batchu et al. [10] observed a similar effect due to transportation stress in goats not previously habituated to transport trailers compared to those habituated.

Several other metabolites indicative of changes in physiological and metabolic statuses were affected by Trt, suggesting that metabolomics could be a powerful tool to gain insights into responses to less intense stressors that traditional methods of assessment (e.g., cortisol) cannot provide. Stress can impact the gut microbiome and, in turn, trimethylamine N-oxide (TMAO) production; therefore, a decreased plasma TMAO concentration in the NH goats may be due to elevated stress. Liu et al. [42] reported that chronic unpredictable stress in rats elevates plasma TMAO and can be associated with depression. The NH goats also had increased plasma hippuric acid concentrations, which are also related to the gut microbiome and oxidative stress [43]. Uric acid levels are associated with anxiety and depression as they modulate hippocampal activity in response to stress [44]. Elevated uric acid concentrations can indicate a stress-induced emotional disturbance in the NH goats. Fumaric acid is a powerful antioxidant and free radical scavenger, and when supplemented in the diet, it has several beneficial effects in Hu sheep [45]. Nevertheless, increased fumaric acid, a key metabolite in cellular oxidative stress response, in the NH group can be a sign of increased stress and the coping mechanism. The increase in blood lactate concentration as a result of increased epinephrine concentration, as seen in the NH goats in this study, is well established. Increases in citrate and α-ketoglutarate in the NH group can also indicate an elevated tricarboxylic acid (TCA) cycle [46], to which lactate can also contribute through conversion to pyruvate. Four of the six acylcarnitines that were influenced by treatment were higher in the NH group, indicating metabolic stress and elevated fatty acid oxidation in mitochondria [47].

Routine handling of goats for veterinary exams had moderate effects on certain metabolites, although there was no differential effect of Trt on the extent of change after the veterinary exam. Plasma glucose concentration was higher at 20 min than at 0 min in the NH goats, indicating the effect of stress-induced epinephrine release. In addition, six of the eight amino acids influenced by the sampling time (lysine, asparagine, proline, threonine, serine, arginine, leucine, isoleucine) were elevated at 20 min. These amino acids are involved in the production of glucose via gluconeogenesis during stress.

Although routine handling for veterinary exams did not cause a significant increase in cortisol concentrations, an accepted measure of the stress response, in either Trt group, there were significant changes in several catecholamines and metabolites. These effects, along with behavioral responses in the NH goats, indicate that these goats were relatively more stressed than the H goats. It is also evident that the blood metabolomic profile of an animal can almost instantly change due to any disturbance to its homeostasis, due to a stressor.

## 5. Conclusions

Regular gentle handling reduced the fear of humans in goats due to habituation to handling. Habituation to handling also increased body weights in meat goats, likely due to decreased excitement and agonistic interactions during feeding time. Goats not accustomed to human handling experienced relatively higher stress and distress levels during a simulated veterinary exam compared to those habituated to handling. Metabolomics proves to be a useful tool in animal welfare research since the metabolome is highly sensitive to physiological and emotional disturbances. The results show that regular human interactions through gentle stroking can improve productivity, reduce the fear of humans, and decrease stress in goats during inevitable management-related handling.

## Figures and Tables

**Table 1:** Pearson correlation analysis among behavioral and physiological stress variables.

Variable	AD	LA	HR	RR	RT	Cortisol	EPI	NorEPI	MN	NorMN
Handled										
ES	−0.0072	−0.1420	0.1736	−0.0399	0.0449	0.2247	0.1219	0.1729	−0.0807	−0.0206
	*0.9670*	*0.4087*	*0.3112*	*0.8171*	*0.7945*	*0.1876*	*0.4786*	*0.3133*	*0.6401*	*0.9049*
AD		−0.6434	−0.0242	−0.0941	0.0353	0.2793	0.1662	0.0554	0.1799	0.2698
		***<0.0001***	*0.8888*	*0.5853*	*0.8381*	*0.0990*	*0.3326*	*0.7481*	*0.2939*	*0.1116*
LA			−0.0881	0.1707	−0.2562	−0.2090	0.1310	−0.1951	−0.2655	−0.2335
			*0.6094*	*0.3195*	*0.1315*	*0.2212*	*0.4463*	*0.2542*	*0.1176*	*0.1706*
Non-handled										
ES	0.0496	−0.0661	−0.1013	0.1416	−0.1625	0.1220	0.4349	0.1216	0.1725	0.3127
	*0.7738*	*0.7018*	*0.5567*	*0.4102*	*0.3437*	*0.4783*	***0.0008***	*0.4798*	*0.3145*	*0.0633*
AD		−0.4311	0.1216	−0.3333	−0.3749	−0.2478	0.1961	−0.2545	0.0502	0.07118
		***0.0087***	*0.4800*	***0.0470***	***0.0243***	*0.1450*	*0.2518*	*0.1341*	*0.7712*	*0.6800*
LA			0.3395	0.3198	0.4199	0.1292	−0.1042	0.3003	−0.0959	−0.1703
			***0.0428***	*0.0572*	***0.0108***	*0.4528*	*0.5455*	*0.0751*	*0.5792*	*0.3205*

Numbers in regular font are correlation coefficients (r-values), and those in italics are probability values. Probability values in bold font indicate significant correlations. ES = exit score; AD = approach distance; LA = latency to approach; HR = heart rate; RR = respiratory rate; EPI = epinephrine; NorEPI = norepinephrine; MN = metanephrine; NorMN = normetanephrine.

**Table 2:** Plasma metabolites in goats significantly affected by treatment (handled (H) vs. non-handled (NH)).

Metabolite	*p*-Value	FDR	Fold Change	Effect Size	Effect Level	Direction of Change
Amino Acids						
Tryptophan	6.18 × 10^−5^	7.22 × 10^−4^	0.86	0.39	Medium	↓ in NH
Histidine	8.37 × 10^−5^	8.78 × 10^−4^	0.85	0.38	Medium	↓ in NH
Aspartic acid	1.90 × 10^−4^	1.81 × 10^−3^	1.30	−0.36	Medium	↑ in NH
Arginine	7.20 × 10^−3^	0.038	0.85	0.26	Small	↓ in NH
Tyrosine	0.015	0.073	0.93	0.23	Small	↓ in NH
Serine	0.019	0.087	0.86	0.23	Small	↓ in NH
Asparagine	0.020	0.087	0.87	0.22	Small	↓ in NH
Glycine	0.030	0.115	1.16	−0.21	Small	↑ in NH
Phosphatidylcholines and Lysophosphatidylcholines						
Diacyl Phosphatidylcholine C38:6	3.72 × 10^−8^	1.95 × 10^−6^	1.32	−0.53	Large	↑ in NH
Diacyl Phosphatidylcholine C40:6	4.54 × 10^−6^	1.03 × 10^−4^	1.17	−0.44	Medium	↑ in NH
Acyl-alkyl Phosphatidylcholine C40:6	7.16 × 10^−6^	1.07 × 10^−4^	1.22	−0.43	Medium	↑ in NH
Diacyl Phosphatidylcholine C38:0	3.33 × 10^−5^	4.38 × 10^−4^	1.19	−0.40	Medium	↑ in NH
Diacyl Phosphatidylcholine C36:6	2.92 × 10^−4^	2.56 × 10^−3^	1.15	−0.35	Medium	↑ in NH
Lysophosphatidylcholine C20:4	8.31 × 10^−4^	5.82 × 10^−3^	1.19	−0.32	Small	↑ in NH
Lysophosphatidylcholine C14:0	0.028	0.114	0.91	0.21	Small	↓ in NH
Acylcarnitines						
Stearylcarnitine (C18)	8.51 × 10^−8^	2.98 × 10^−6^	1.54	−0.52	Large	↑ in NH
Acetylcarnitine (C2)	4.90 × 10^−6^	1.03 × 10^−4^	1.36	−0.44	Medium	↑ in NH
Palmitoylcarnitine (C16)	7.19 × 10^−4^	5.39 × 10^−3^	1.21	−0.33	Small	↑ in NH
Propionylcarnitine (C3)	2.28 × 10^−3^	0.014	0.87	0.29	Small	↓ in NH
Palmitoleylcarnitine (C16:1)	6.87 × 10^−3^	0.038	1.06	−0.26	Small	↑ in NH
Carnitine (C0)	0.041	0.154	0.90	0.20	Small	↓ in NH
Organic Acids						
Uric acid	6.57 × 10^−6^	1.07 × 10^−4^	1.39	−0.44	Medium	↑ in NH
Fumaric acid	5.81 × 10^−4^	4.69 × 10^−3^	1.17	−0.33	Medium	↑ in NH
Lactic acid	9.86 × 10^−4^	6.47 × 10^−3^	1.65	−0.32	Small	↑ in NH
alpha-Ketoglutaric acid	4.12 × 10^−3^	0.024	1.16	−0.28	Small	↑ in NH
Citric acid	0.026	0.110	1.11	−0.21	Small	↑ in NH
Other Metabolites						
Trimethylamine N-oxide	3.28 × 10^−10^	3.44 × 10^−8^	0.23	0.61	Large	↓ in NH
Hippuric acid	8.71 × 10^−3^	0.044	1.35	−0.25	Small	↑ in NH

**Table 3:** Plasma metabolites in goats significantly affected by sampling time (before routine physical exam (0 min) vs. after physical exam (20 min)).

Metabolite	*p*-Value	FDR	Fold Change	Effect Size	Effect Level	Direction of Change
Amino acids						
Lysine	7.88 × 10^−5^	1.70 × 10^−3^	0.86	0.20	Small	↓ in 20 min
Asparagine	1.09 × 10^−4^	1.91 × 10^−3^	0.91	0.19	Small	↓ in 20 min
Proline	3.20 × 10^−4^	3.36 × 10^−3^	0.93	0.19	Small	↓ in 20 min
Threonine	3.20 × 10^−4^	3.36 × 10^−3^	0.84	0.22	Small	↓ in 20 min
Serine	8.75 × 10^−4^	7.07 × 10^−3^	0.84	0.17	Small	↓ in 20 min
Arginine	1.22 × 10^−3^	9.17 × 10^−3^	0.85	0.19	Small	↓ in 20 min
Leucine	1.72 × 10^−3^	0.012	0.94	0.16	Small	↓ in 20 min
Isoleucine	2.08 × 10^−3^	0.014	0.95	0.16	Small	↓ in 20 min
Other metabolites						
Succinic acid	3.47 × 10^−6^	2.38 × 10^−4^	0.89	0.25	Small	↓ in 20 min
Lactic acid	4.53 × 10^−6^	2.38 × 10^−4^	0.67	0.17	Small	↓ in 20 min
Glucose	1.49 × 10^−4^	2.23 × 10^−3^	1.06	−0.29	Small	↑ in 20 min
Pyruvic acid	7.99 × 10^−4^	6.99 × 10^−3^	1.09	−0.19	Small	↑ in 20 min
Total dimethylarginine	0.026	0.103	0.93	0.17	Small	↓ in 20 min

## Data Availability

The data presented in this study are available on request from the corresponding author.
